# A Non-Invasive Continuous Respiration Rate Monitoring Device for Dairy Cattle Under Commercial Farm Conditions

**DOI:** 10.3390/ani16060984

**Published:** 2026-03-21

**Authors:** Mathias Eisner, Manuel Jedinger, Daniel Eingang, Manuel Raggl, Manuel Frech, Peter Lenzelbauer, Michael Harant, Oliver Orasch, Philipp Breitegger

**Affiliations:** 1 smaXtec Animal Care GmbH, Sandgasse 36/2, A-8010 Graz, Austria; manuel.jedinger@smaxtec.com (M.J.); peter.lenzelbauer@smaxtec.com (P.L.); oliver.orasch@smaxtec.com (O.O.); philipp.breitegger@smaxtec.com (P.B.); 2 Institute of Livestock Research, Agricultural Research and Education Centre Raumberg-Gumpenstein, Alt-Irdning 11, A-8952 Irdning-Donnersbachtal, Austria

**Keywords:** respiration rate, dairy cattle, acoustic monitoring, wearable sensor, animal welfare, long-term monitoring

## Abstract

Respiration rate (RR) is a key physiological indicator of health, stress, and thermoregulatory load in dairy cattle, yet continuous RR monitoring under commercial farm conditions remains challenging. In this Technical Note, we present a non-invasive clip-on nose ring device for continuous respiration monitoring based on acoustic recording directly at the nostril. The device integrates a MEMS microphone, embedded electronics, battery, and removable storage in a sealed, mechanically robust housing suitable for real-world barn environments. The system was deployed on five dairy cows under commercial farm conditions, enabling repeated multi-day recordings over several weeks. The respiration rate was extracted offline from raw audio using a deterministic signal-processing pipeline based on multiscale periodicity detection. Algorithm-derived RR estimates were evaluated against manually annotated breath events. Using 10-min rolling median values, the algorithm achieved a mean absolute error (MAE) of 1.47 breaths per minute (bpm), a root mean square error (RMSE) of 1.92 bpm, and a high correlation with reference values (r = 0.98, R^2^ = 0.96). In addition to short-term accuracy, the system enabled stable multi-day monitoring. Group-level analysis across all five animals revealed a clear diurnal respiration pattern over multiple consecutive days, with lower RR during nighttime and higher RR during daytime summer conditions, without signs of a baseline drift. These results demonstrate the feasibility of continuous, long-term respiration monitoring in dairy cattle using an audio-based clip-on nose ring device and provide a practical foundation for longitudinal (multi-day, within-animal) RR assessment under commercial farm conditions, with potential for future extensions towards advanced respiratory health monitoring. While the system demonstrated stable performance under summer farm conditions, validation under extreme heat-stress environments and larger animal cohorts is required for comprehensive population-level assessment.

## Simple Summary

Breathing rate is an important sign of health, stress, and heat strain in dairy cows, but it is usually measured by visually counting body movements for short periods of time. This approach is labor-intensive and cannot provide continuous information. Camera-based systems may require special installation, stable lighting, or controlled positioning of animals, which limits their use on commercial farms. In this study, we developed a small clip-on nose ring device that records breathing sounds directly at the nostril. The device can be easily attached without modifying the barn or using external equipment, and it functions during normal daily activities such as feeding and drinking without disturbing the cow. We tested the system under real farm conditions over several weeks and found that it provided accurate breathing rate measurements and stable long-term recordings. The system also revealed clear daily breathing patterns. This practical and easy-to-use approach may support farmers and veterinarians in monitoring animal health, improving welfare, and detecting problems earlier under everyday farm conditions.

## 1. Introduction

Respiration rate (RR) is a fundamental physiological indicator widely used to assess the health, stress status, and welfare of dairy cattle [1,2,3]. RR reflects changes in thermoregulatory load, respiratory function, and general stress physiology, and variations in RR have been linked to environmental conditions, production status, and animal comfort in commercial herds [2,3,4]. Recent large-scale analyses in dairy cattle have shown that RR can vary substantially depending on environmental and animal-related factors, and that commonly applied THI thresholds, which are often used as proxies for heat stress severity, may inadequately capture the magnitude and variability of the respiration rate response observed under practical farm conditions [2,3].

Despite its importance, routine RR monitoring remains challenging because commonly used measurement approaches are inconsistent and labor-intensive. The most frequently applied method in research and on-farm practice is often visual observation and manual counting of flank movements over short time windows [5]. In addition to being limited to short observation windows, manual counting is time-consuming and therefore typically restricted to a limited number of animals, difficult to scale to large herds, and prone to intra- and inter-observer variability. Furthermore, a lack of standardized counting definitions and protocols reduces comparability between studies and may introduce systematic bias in RR estimates. A recent review emphasized that different counting methods (e.g., fixed time windows versus counting a fixed number of breaths) can yield different RR values which underlines the need for improved and standardized approaches [5].

Automated RR measurement technologies have been proposed, including camera-based systems, infrared thermography, and other sensor-based approaches [6,7,8,9,10]. While these methods can reduce human effort and provide objective measurements, they often depend on stable animal positioning, unobstructed line-of-sight, or controlled environmental conditions, which can be difficult to ensure under commercial farm conditions. Furthermore, long-term continuous RR monitoring remains technically challenging due to noise, movement artifacts, and practical constraints on sensor placement and durability in real barn environments.

Given these limitations, there is a clear need for a robust, practical method capable of continuous RR monitoring with minimal interference to normal animal management. In this Technical Note, we present a novel clip-on nose ring device equipped with a high-sensitivity microphone that continuously records respiration-related acoustic signals inside the nostrils. The device logs data to an onboard SD card and is rechargeable, enabling autonomous operation over days to weeks. We describe the device design, data acquisition concept, and demonstrate its feasibility for reliable long-term RR monitoring in dairy cattle under commercial barn conditions.

## 2. Materials and Methods

### 2.1. Animals and Study Design

Five dairy cows (1 Fleckvieh, 4 Holstein) were equipped with the disclosed clip-on respiration monitoring device. Data collection was performed under real-world dairy barn conditions during normal herd management at the Raumberg-Gumpenstein research dairy facility (Austria). The herd was housed in a loose housing system consisting of an indoor resting and feeding area with access to an outdoor open area. Cows had free access to water and to an automated concentrate feeding station, and received a total mixed ration at the feed bunk during routine feeding times. Animals could freely move between indoor and outdoor areas throughout the study period. No changes to routine housing or management were introduced for the purpose of this study.

Ambient temperature and relative humidity data from a nearby weather station (~200 m from the barn) were reviewed to characterize general environmental conditions during the study period. In-barn microclimate conditions were not directly recorded, and no formal THI-based analysis was performed in this feasibility study.

To assess long-term feasibility and robustness, repeated multi-day recordings were conducted for each animal. In total, approximately 16 recording days per animal—four trial phases with four consecutive days each—of recordings were collected: the devices were mounted Monday–Friday, removed on Friday for SD card readout and recharging, and mounted again on Monday, repeated over roughly 4 weeks.

During device deployment, animals were visually inspected daily by farm personnel for signs of discomfort, nasal irritation, discharge, or behavioral aversion. Devices were removed weekly for charging and data retrieval, allowing inspection of the nostril and surrounding tissue. In addition, routinely collected herd-management metrics, including daily water intake and rumination time, were monitored throughout the observation period to detect potential deviations from normal feeding-related behavior. These parameters were derived from the farm’s routinely operating smaXtec intraruminal bolus monitoring system, which continuously records individual water intake and rumination time as part of standard herd management. The boluses were not administered for the purpose of this study and were already present in the herd prior to device deployment. All cows in the comparison (trial and non-trial animals) were monitored using the same system.

### 2.2. Device Design and Manufacturing

**Figure 1:**
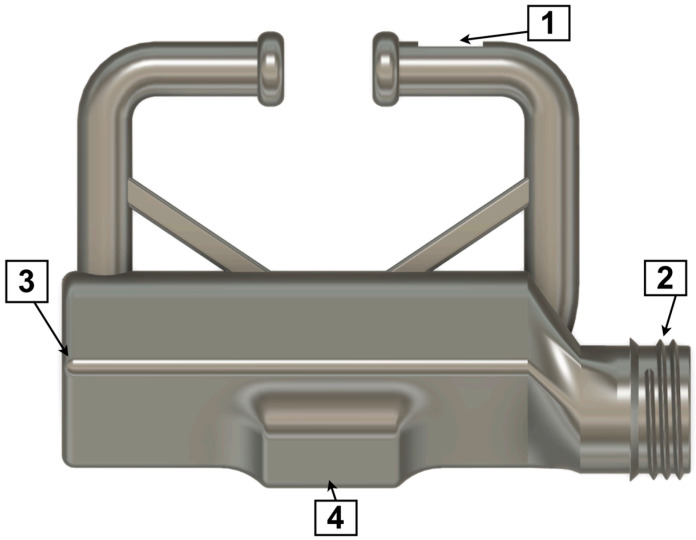
Clip-on nose ring respiration monitoring device. Numbered labels indicate key components: (1) MEMS microphone positioned for nostril audio capture; (2) screw-on lid providing access to USB-C charging port and SD card slot; (3) housing containing battery and embedded electronics; (4) magnet position used for activation/deactivation of logging (magnet attached = logging disabled; magnet removed = logging enabled). Overall device dimensions in the shown view: 87 mm (height, bottom-to-top) × 122 mm (width, left-to-right); device thickness: 28 mm (side view).

**Figure 2:**
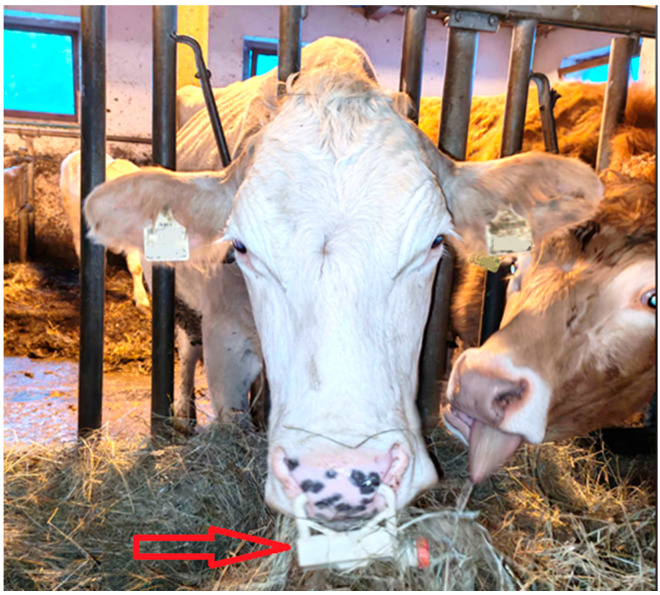
Clip-on nose ring respiration monitoring device (marked by arrow) mounted on a cow during normal barn activity. The device remains attached during routine behaviors such as feeding, demonstrating unobtrusive integration and suitability for use under practical farm conditions.

The respiration monitoring device consists of a clip-on nose ring assembly incorporating a microphone, embedded electronics, battery, and onboard storage (Figure 1). The micro-electromechanical systems (MEMS) microphone (Figure 1, Label 1) is mounted on the upper part of the clip and positioned approximately 2–3 cm inside one nostril, oriented upward to capture airflow-related breath sounds. To protect the acoustic inlet from water and dirt while maintaining sound transmission, the microphone opening is covered with an expanded polytetrafluoroethylene (ePTFE) acoustic membrane. Battery and electronics are housed in the lower portion of the clip (Figure 1, Label 3). A screw-on lid provides access to the USB-C charging interface and SD card slot for data retrieval (Figure 1, Label 2). Device activation and deactivation are controlled via a magnetic switch, with the magnet placement indicated in Figure 1 (Label 4). The entire assembly is encapsulated (potted) in resin using a dedicated mold, resulting in a sealed and mechanically stable construction. This design protects internal components from moisture exposure, oxidation, and mechanical damage, and enables reliable operation under barn conditions including contact with dirt and water (e.g., during drinking). Figure 2 illustrates the device mounted on a cow during normal barn activity, demonstrating stable attachment and unobtrusive operation during routine behaviors such as feeding.

### 2.3. Device Operation and Data Acquisition

Device operation is controlled via a magnetic switch mechanism: when a magnet is attached to the device, logging is disabled; removing the magnet initiates audio recording. This allows simple activation and deactivation during handling without requiring external tools or wireless connectivity. Once activated, the device records raw audio directly at the animal’s nostril and stores the recordings locally on an SD card.

Audio data were stored as WAV files with a sampling frequency of 8 kHz. This sampling rate was selected as a compromise between storage efficiency—enabling multi-day recordings on multi-GB SD cards—and interpretability, as respiration sounds remained clearly audible for manual inspection and expert review. Devices were periodically removed according to the study schedule (see Section 2.1) for SD card data extraction and battery recharging.

### 2.4. Manual Annotation of Exhalation Events

**Figure 3:**
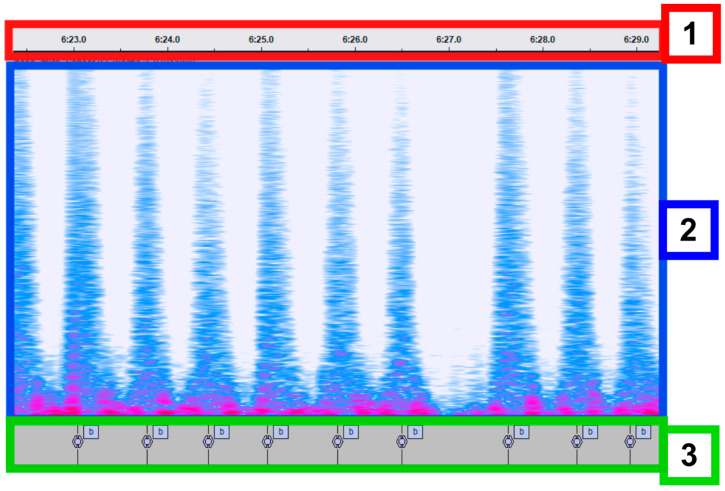
Manual breath annotation workflow using Audacity. A representative ~5 s audio segment recorded at the nostril is shown. (1) Timeline showing seconds. (2) audio segment recorded from nose clip showing exhalation sounds as peaks. (3) hand annotations, each ‘b’ denotes an exhalation sound.

To generate reference labels for algorithm development and evaluation, approximately 7 h of audio recordings were manually annotated by trained personnel using Audacity (Audacity Team, version 3.7.6) [11]. Breath events were identified based on clearly audible respiration sounds, which were dominated by expiratory exhalation events due to microphone placement near the nostrils. Figure 3 illustrates the manual annotation workflow, showing a representative audio segment (Figure 3, Label 1) alongside its frequency spectrogram (Figure 3, Label 2) and the corresponding label track containing manually annotated exhalation events (Figure 3, Label 3). Individual annotated snippets were additionally reviewed by a veterinarian to confirm agreement in ambiguous segments and to ensure physiological plausibility of the interpreted respiration events.

### 2.5. Respiration Rate Extraction from Audio Recordings

**Figure 4:**
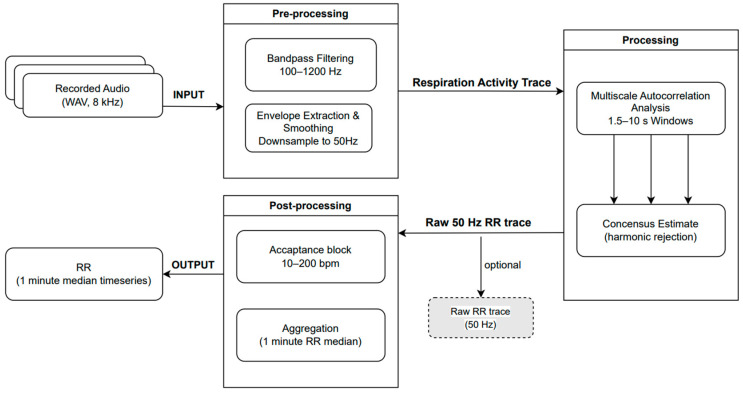
Schematic overview of the respiration rate (RR) extraction pipeline from nostril audio recordings. Raw audio (WAV, 8 kHz) is bandpass-filtered and transformed into a respiration activity trace via energy extraction and low-pass smoothing, followed by down-sampling. RR is estimated by detecting periodicity using rolling autocorrelation across multiple window sizes. Candidate breathing periods are converted to breaths per minute and combined to obtain a robust consensus RR estimate within a physiologically plausible range, which is summarized as median values over 1 min intervals for reporting.

Figure 4 provides an overview of the signal-processing pipeline used to extract respiration rate (RR) from nostril audio recordings. RR was extracted offline from the recorded audio using a deterministic signal-processing pipeline implemented in Python (version 3.10.16, Python Software Foundation, Wilmington, DE, USA). The pipeline does not involve machine learning, parameter fitting, or training on labeled data; all processing steps and parameters were predefined based on signal characteristics and physiological plausibility. Raw audio (WAV, 8 kHz) was first bandpass filtered (100–1200 Hz) and transformed into a respiration activity trace via energy extraction (envelope extraction) and low-pass smoothing (2 Hz), followed by down-sampling to 50 Hz.

Periodicity in the respiration activity trace was detected using a rolling autocorrelation approach evaluated over multiple window sizes (1.5–10 s). This range was selected to ensure that several consecutive breath cycles were contained within each analysis window across physiologically plausible respiration rates. Shorter windows increase sensitivity to faster breathing frequencies, whereas longer windows improve stability for slower respiration rates and reduce susceptibility to transient noise. Evaluating multiple window lengths therefore enhances robustness across varying respiratory dynamics. Candidate breathing periods were converted to breaths per minute and combined across window sizes to obtain a robust consensus RR estimate within a physiologically plausible range (acceptance range: 10–200 breaths per minute). For reporting, RR timeseries were summarized as median values over 10 min intervals; higher temporal resolution outputs can be generated if required.

### 2.6. Embedded Logging and Data Processing

The embedded firmware controls audio sampling, power management, and writing WAV files to an SD card. All respiration rate processing was performed offline. Raw audio recordings remain available for replay and audit, allowing expert review of uncertain segments and additional downstream analyses, e.g., veterinary assessment of abnormal breathing sounds.

## 3. Results

### 3.1. Field Deployment and Data Yield

The clip-on respiration monitoring device was successfully deployed on five dairy cows under real-world barn conditions. Across all animals and deployment periods, a total of 59,280 min (≈988 h) of nostril audio were recorded, corresponding to repeated multi-day recordings per cow over several weeks. Device attachment, activation via magnetic switch, and SD-based data logging functioned reliably throughout the study period, with no systematic data loss observed during routine operation.

From the full dataset, a subset of recordings was selected for detailed manual annotation to generate reference labels for algorithm evaluation. In total, 420 min (7 h) of audio tracks were manually annotated by trained personnel, with annotations distributed across multiple animals and recording days. Selected annotated segments were additionally inspected by a veterinarian to confirm physiological plausibility of respiration events in ambiguous cases.

Table 1 summarizes the total recorded audio duration and manually annotated duration for each subject. The labeled data covers a range of animals and recording conditions, providing a representative basis for evaluating respiration rate extraction performance under practical farm conditions.

### 3.2. Agreement Between Algorithm Output and Expert Annotations

Quantitative agreement between algorithm-derived respiration rate estimates and expert annotations was evaluated using 10-min rolling median respiration rate values. Manual annotations were available for two animals, selected to provide long, continuous recording segments. This approach was selected due to the highly time-intensive nature of manual annotation of high-frequency data, with priority given to longer labelled time spans rather than a larger number of short segments from additional animals. The annotated dataset was used exclusively for quantitative accuracy assessment. Multi-day respiration rate time series were computed for all five animals, with representative excerpts shown to illustrate longitudinal stability.

**Figure 5:**
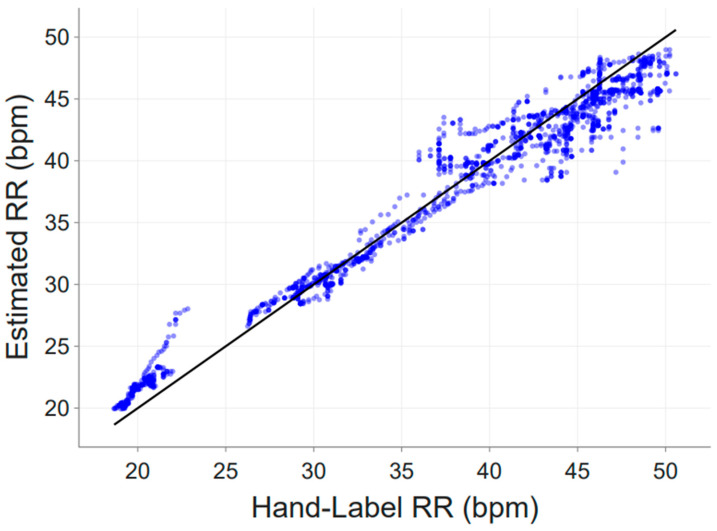
Comparison of algorithm-derived respiration rate (RR) estimates with manually annotated reference RR values. Each point represents a paired 10-min rolling median value. The solid line denotes the line of identity (y = x), indicating very strong agreement (r = 0.98, R^2^ = 0.96) between methods.

In total, 1774 observations were obtained from two sensors (Subject 2 and Subject 4). Figure 5 shows the scatter plot of algorithm-derived respiration rate versus manually annotated reference values. Data points clustered closely around the line of identity across the observed respiration rate range, indicating good overall agreement.

Summary performance metrics are reported in Table 2. Across all paired observations, the mean absolute error (MAE) was 1.47 breaths per minute (bpm), with a root mean square error (RMSE) of 1.92 bpm. The mean bias (estimate − label) was −0.29 bpm, indicating a minimal systematic underestimation of respiration rate by the algorithm. The standard deviation of the error was 1.90 bpm. A very strong linear relationship was observed between estimated and labeled respiration rates (r = 0.98; R^2^ = 0.96), indicating close agreement across the observed respiration rate range.

Results were comparable between the two sensors individually, with MAE values of 1.42 bpm (Subject 2) and 1.52 bpm (Subject 4), and no pronounced systematic deviation observed.

### 3.3. Multi-Day Group Respiration Rate Dynamics Under Farm Conditions

**Figure 6:**
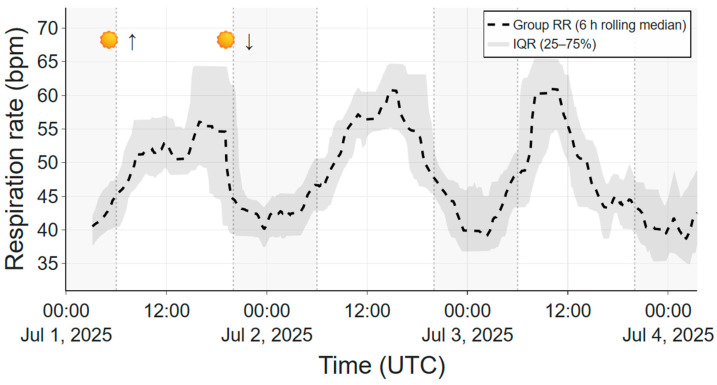
Group respiration rate across five cows over 3.5 days, shown as a dashed 6-h rolling median. The shaded band indicates variability in the underlying 10-min averaged group respiration rate (interquartile range (IQR), 25th–75th percentile). A clear diurnal pattern is visible, with lower respiration rates at night and higher values during daytime under summer conditions. Sun symbols with arrows indicate approximate sunrise and sunset times for visual orientation of the day night cycle.

To illustrate long-term robustness under practical farm conditions, Figure 6 shows ~3.5 days of the group RR computed across five animals and summarized using a 6-h rolling median. A clear diurnal pattern is visible, with lower RR during nighttime and higher RR from late morning to early afternoon, consistent with summer conditions. The absence of baseline drift or abrupt shifts over multiple day–night cycles demonstrates the suitability of the system for continuous multi-day monitoring under real-world farm conditions.

### 3.4. Welfare-Relevant Herd Metrics During Device Deployment

To assess potential behavioral impacts of device wear, routinely recorded herd-management metrics were evaluated over the study period. Median daily water intake and rumination index of instrumented cows remained within the interquartile range observed in contemporaneous herd mates housed under the same conditions (Table 3). No abrupt reductions or progressive declines were observed during the observation period. These descriptive findings do not indicate marked disruption of feeding or rumination behavior associated with device deployment.

### 3.5. Failure Modes and Limitations

The high audio sampling rate of the system generates a large volume of raw data (e.g., continuous 8 kHz WAV recordings), which poses practical challenges for downstream processing and data management.

Periods of cow vocalization, e.g., mooing, or strong environmental noise can temporarily mask the respiratory signal, preventing respiration rate extraction during these intervals. Such events were observed infrequently and typically affected only short time segments.

Ongoing work focuses on migrating the algorithm directly onto the sensors to enable on-device RR extraction and wireless transmission of the results rather than storing raw audio data. Furthermore, although recordings were obtained under summer conditions, the study did not include controlled exposure to extreme heat-stress environments. Algorithm performance under severe heat stress and very short inter-breath intervals warrants further investigation.

Lastly, we comment on the limitations introduced by manually annotated reference data. Although exhalation events were clearly audible in most recordings and annotations were reviewed by trained personnel and a veterinarian, manual labeling inherently involves temporal uncertainty. Small deviations in the placement of breath event markers (e.g., slightly earlier or later annotation of expiratory sounds) can propagate into respiration rate estimates, particularly when RR is derived from short inter-breath intervals. This introduces an irreducible source of variability that is independent of algorithm performance and is also present in conventional manual RR counting methods. Consequently, part of the observed estimation error likely reflects uncertainty in the reference labels rather than true algorithmic inaccuracy

## 4. Discussion

Continuous respiration rate (RR) monitoring in dairy cattle is highly relevant for assessing animal welfare, thermoregulatory load, and respiratory health [1,2,3,12], yet remains difficult to implement under commercial farm conditions. Manual RR assessment by visual flank counting is labor-intensive, subjective, and typically limited to short observation windows, which may not capture prolonged or transient changes in respiratory dynamics, and is prone to observer-related variability [7].

In this Technical Note, we present a clip-on nose ring device that enables continuous, multi-day recording of respiration-related acoustic signals directly at the nostril. The device design proved suitable for practical farm use, as demonstrated by repeated multi-day deployments across five cows over several weeks, including stable operation during routine behaviors such as feeding and drinking. The sealed construction and protected microphone inlet allowed reliable data acquisition under conditions involving dirt, moisture, and animal movement. While no marked deviations in routinely collected feeding-related metrics were observed, formal behavioral scoring (e.g., grooming, social interactions) and detailed clinical lesion scoring were beyond the scope of this technical feasibility study. Dedicated welfare-focused investigations under controlled conditions will be required to comprehensively evaluate long-term behavioral and physiological impact.

In contrast to approaches that estimate respiration indirectly from body or head movements, the presented system captures airflow-related acoustic signals directly at the nostril, enabling direct detection of breath events independent of gross body motion. Unlike camera-based systems, no line-of-sight, controlled positioning, or dedicated barn infrastructure is required. This configuration therefore combines direct physiological signal acquisition with infrastructure-independent deployment under commercial farm conditions.

Quantitative evaluation against manually annotated reference data showed strong agreement between algorithm-derived and expert-labeled respiration rates. Using paired 10-min rolling median values, the algorithm achieved a mean absolute error of 1.47 bpm and a root mean square error of 1.92 bpm across two sensors, with high correlation (r = 0.98, R^2^ = 0.96). These error magnitudes are within the range of variability reported for manual RR assessment, which itself is subject to observer-dependent temporal uncertainty [7]. Moreover, the observed errors are small relative to physiologically relevant RR changes observed under heat stress or disease-related conditions in dairy cattle [1,2,3,12]. This indicates that the proposed approach can provide meaningful and reliable RR estimates under real-world conditions.

Beyond short-term accuracy, the ability to capture long-term trends is a key advantage of the presented system. Multi-day group-level analysis revealed a consistent diurnal respiration pattern, with lower RR during nighttime and higher values during daytime summer conditions, consistent with known environmental influences on respiration in dairy cattle [2,3]. The absence of baseline drift or abrupt shifts over multiple day–night cycles supports the suitability of the system for longitudinal monitoring. Group-level aggregation and temporal smoothing further reduce sensitivity to transient artifacts while preserving biologically meaningful dynamics, making this approach well suited for studying environmental and management-related influences on respiration.

A further strength of the audio-based modality is that it captures rich respiratory information beyond respiration rate alone. In addition to exhalation-exhalation timing, the recorded signals contain information on breath intensity, duration, and spectral characteristics, which may be indicative of respiratory effort or abnormal breathing patterns. Moreover, other physiological sounds, such as ructus (eructation), are audible in the recordings but were not analyzed in the present work. These additional signal components were intentionally left out of scope here, but they represent promising targets for future machine learning–based analyses aimed at detecting respiratory disorders or broader health-related conditions.

The present study focused on controlled multi-day validation in a limited cohort (number of animals = 5) to demonstrate technical feasibility and signal robustness under commercial farm conditions. Broader population-level validation across diverse herds and environmental contexts represents a logical next step.

Several limitations should be considered. The high sampling rate of continuous audio recording generates large data volumes, requiring periodic handling for data retrieval and battery recharging. In addition, periods of vocalization or strong environmental noise can temporarily mask respiratory sounds, leading to short gaps in RR estimation. These effects were observed infrequently and did not compromise long-term trends but should be considered when interpreting high-resolution outputs. Ongoing development focuses on embedded, on-device RR extraction and wireless data transmission to reduce data volume and handling requirements.

## 5. Conclusions

This Technical Note demonstrates the feasibility of a non-invasive, clip-on nose ring device for continuous respiration rate monitoring in dairy cattle under commercial farm conditions. The system provides accurate RR estimates compared with expert annotations and enables stable multi-day measurements that capture physiologically meaningful diurnal patterns. By combining robust hardware design with deterministic signal processing, the approach offers a practical foundation for further development and validation of long-term respiratory monitoring approaches. Future developments will focus on embedded processing and the exploitation of richer acoustic features for advanced health and welfare applications.

## Figures and Tables

**Table 1:** Data summary per subject.

Subject Nr.	Audio Minutes	Hand Annotated Minutes
1	8910	
2	11,670	180
3	12,990	
4	13,080	240
5	12,630	
Total	59,280	420

**Table 2:** Performance metrics for algorithm-derived respiration rate estimates compared with manually annotated reference values, reported per subject and overall. The number of evaluated exhalation events is denoted with *n*.

Subject Nr.	*n*	MAE (bpm)	RMSE (bpm)	Bias (bpm)	SD-Error (bpm)	r	R^2^
2	885	1.42	1.97	−1.11	1.63	0.98	0.93
4	889	1.52	1.87	0.52	1.80	0.99	0.97
Total		1.47	1.92	−0.29	1.90	0.98	0.96

**Table 3:** Herd management metrics.

	Number of Cows	Number of Cow Days	Water Median, (IQR) (L)	Rumination Median, (IQR) (Minutes)
Trial Cows	5	150	96.1, (83.9–107.4)	564.5, (539.8–586.1)
Herd Cows (excl. trial)	44	1303	85.8, (73.6–101.3)	561.1, (525.2–587.7)

## Data Availability

The data presented in this study are not publicly available due to the very large amount of raw audio recordings and considerations related to animal handling. Processed respiration rate time series and derived summary data are available from the corresponding author upon reasonable request.
